# Expression of Nrf2 and NF-κB transcription factors in breast cancer and breast fibroadenoma: Insights for a new therapeutic approach

**DOI:** 10.18632/oncotarget.27574

**Published:** 2020-05-05

**Authors:** Camila Maria Simplicio-Revoredo, Renato de Oliveira Pereira, Mariella de Almeida Melo, Pedro Vitor Lopes-Costa, Paulo de Tarso Moura-Borges, Emerson Brandão Sousa, Fidelis Manes Neto, Viriato Campelo, Ione Maria Ribeiro Soares-Lopes, Maria da Conceição Barros-Oliveira, Cleciton Braga Tavares, Alesse Ribeiro dos Santos, Camila Guedes Borges de Araújo, Eid Gonçalves Coelho, Larysse Cardoso Campos-Verdes, Aldenora Oliveira do Nascimento-Holanda, Jackeline Lopes Viana, Maria Liduina Meneses Bezerra-Chaves, Rodrigo José de Vasconcelos-Valença, Lina Gomes dos Santos, Lauro Rodolpho Soares-Lopes, André Luiz Pinho-Sobral, Luiz Henrique Gebrim, Benedito Borges da Silva

**Affiliations:** ^1^ Postgraduate Program, Northeast Biotechnology Network (RENORBIO), Department of Health, Federal University of Piauí, Teresina, Piauí 64000-020, Brazil; ^2^ Postgraduate Program in Health Sciences, Department of Mastology, Federal University of Piauí, Teresina, Piauí 64000-020, Brazil; ^3^ Getúlio Vargas Hospital, Department of Mastology, Federal University of Piauí, Teresina, Piauí 64000-020, Brazil

**Keywords:** breast cancer, fibroadenoma, NRF2, NF-κB, immunohistochemistry

## Abstract

**Background:** Cancer and fibroadenoma are the most common breast tumors in women of reproductive age. Nuclear factor erythroid 2-related factor 2 (Nrf2) and the nuclear factor kappaB (NF-κB) transcription factor play an important role in the inflammatory process and in cell proliferation. However, few studies have analyzed these markers in breast cancer and fibroadenoma in women of reproductive age.

**Results:** Light microscopy showed a higher concentration of anti-Nrf2 and anti-NF-κB-stained nuclei in breast cancer than in fibroadenoma. The mean percentage of stained nuclei for Nrf2 was 7.12 ± 5.2 and 43.21 ± 19.83 in the control and study groups, respectively (p < 0.0001). The mean percentage of anti-NF-κB was 10.75 ± 7.09 and 56.14 ± 21.19 (mean ± standard deviation) in the control and study groups, respectively (p < 0.0001). Histological grade 3 tumors showed a significantly higher expression of Nrf2 and NF-κB than grade 1 tumors (p < 0.05).

**Material and methods:** This study was approved by the Institutional Review Board of Federal University of Piaui and all patients assigned an inform consent term prior to the study initiation. Nrf2 and NF-κB expression was evaluated by immunohistochemistry in 66 patients, divided into two groups, control (fibroadenoma, n = 36) and study (cancer, n = 30). The data were analyzed using ANOVA test and the statistical significance was established at p < 0.05.

**Conclusion:** Nrf2 and NF-κB expression was significantly higher in breast cancer than in fibroadenoma, in addition to having a greater association with more aggressive tumors.

## INTRODUCTION

Breast cancer is the most commonly diagnosed malignant neoplasm in women worldwide and, although most prevalent in postmenopausal women, it is estimated that 4.8–5% of cases occur in young adults under 40 years of age [1]. On the other hand, mammary fibroadenoma is the most common benign lesion in young women of reproductive age, being considered by some as not a tumor, but only an aberration of normal development and involution (ANDI) [2–3]. Despite some controversy over a higher risk for breast cancer in the presence of fibroadenoma, according to the concept of ANDI, this lesion may be considered as a control for breast cancer biomarker studies [4–8].

Despite early diagnosis through mammographic screening, and therapeutic advances, breast cancer is associated with high mortality, requiring a change in strategy when one therapy is ineffective [8]. Changes in therapeutic strategy may be suggested by biomarkers, since alterations induced in biomolecular markers by the administration of drugs linked to the cell proliferation and apoptosis for shorter period of time (2 to 4 weeks) ocurr before any clinical response of the tumor to the treatment [9, 10]. Likewise, nuclear factor erythroid 2-related factor 2 (Nrf2) and nuclear factor kappa B (NF-κB) transcription factor, related to cell proliferation and apoptosis are associated to malignant progression, poor prognosis and therapy resistance and thus may also suggest therapy changes [11, 12].

The Nrf2 pathway represents one of the most important cellular defensive mechanism against xenobiotic/electrophilic and oxidative stress, however the aberrant activation or accumulation of Nrf2 has recently been found to promote cancer development, progression and therapy resistance [11, 13]. There is increasing evidence to show the complexity of Nrf2 function, in addition to its antioxidant and detoxifying response, implicating it in many other molecular processes including inflammatory responses, metabolic reprogramming, cell proliferation, senescence and survival [14].

The NF-κB signaling pathway includes a family of transcription factors that play a role in immunity, inflammation and various cancers, including breast cancer [15]. Activation of NF-κB leads to the induction of target genes that may inhibit apoptosis, interaction with cell cycle regulation, cell invasion, contribute to tumorigenesis, inflammation and metastatic growth, as well as resistance to radio and chemotherapy [12].

However, there are few studies evaluating the immunohistochemical expression of Nrf2 and NF-κB in women with breast cancer, and to the best of our knowledge no published research has simultaneously evaluated the expression of these biomarkers in women of reproductive age with breast cancer and fibroadenoma. This led us to design of the present study comparing Nrf2 and NF-κB expression in women of reproductive age with breast cancer and fibroadenoma.

## RESULTS

**Figure 1:**
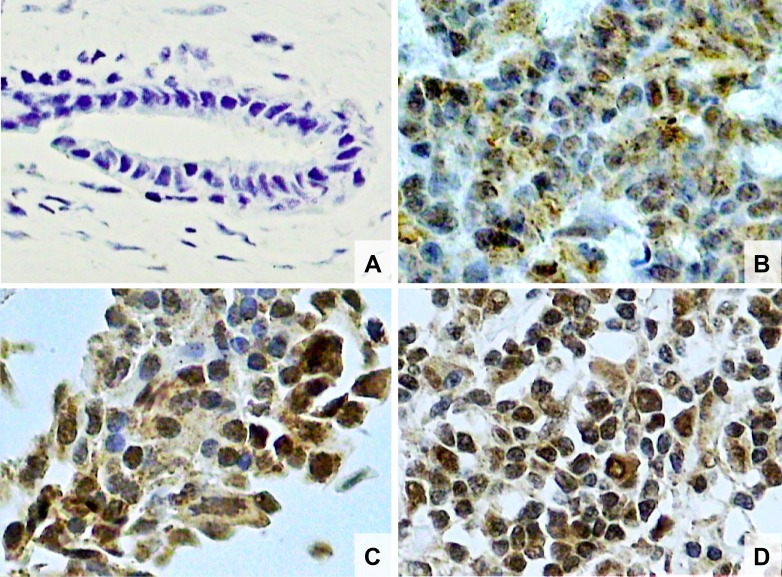
Photomicrographs of histological sections of breast fibroadenoma (**A**), histological grade 1 breast cancer (**B**), grade 2 breast cancer (**C**) and grade 3 breast cancer (**D**). Note the higher concentration of Nrf2 stained nuclei in breast cancer cells compared to fibroadenoma cells and also a higher concentration of Nrf2 stained nuclei in histological grade 3 than grade 2 and higher in grade 2 than grade 1 breast cancer (original magnifcation × 400).

**Figure 2:**
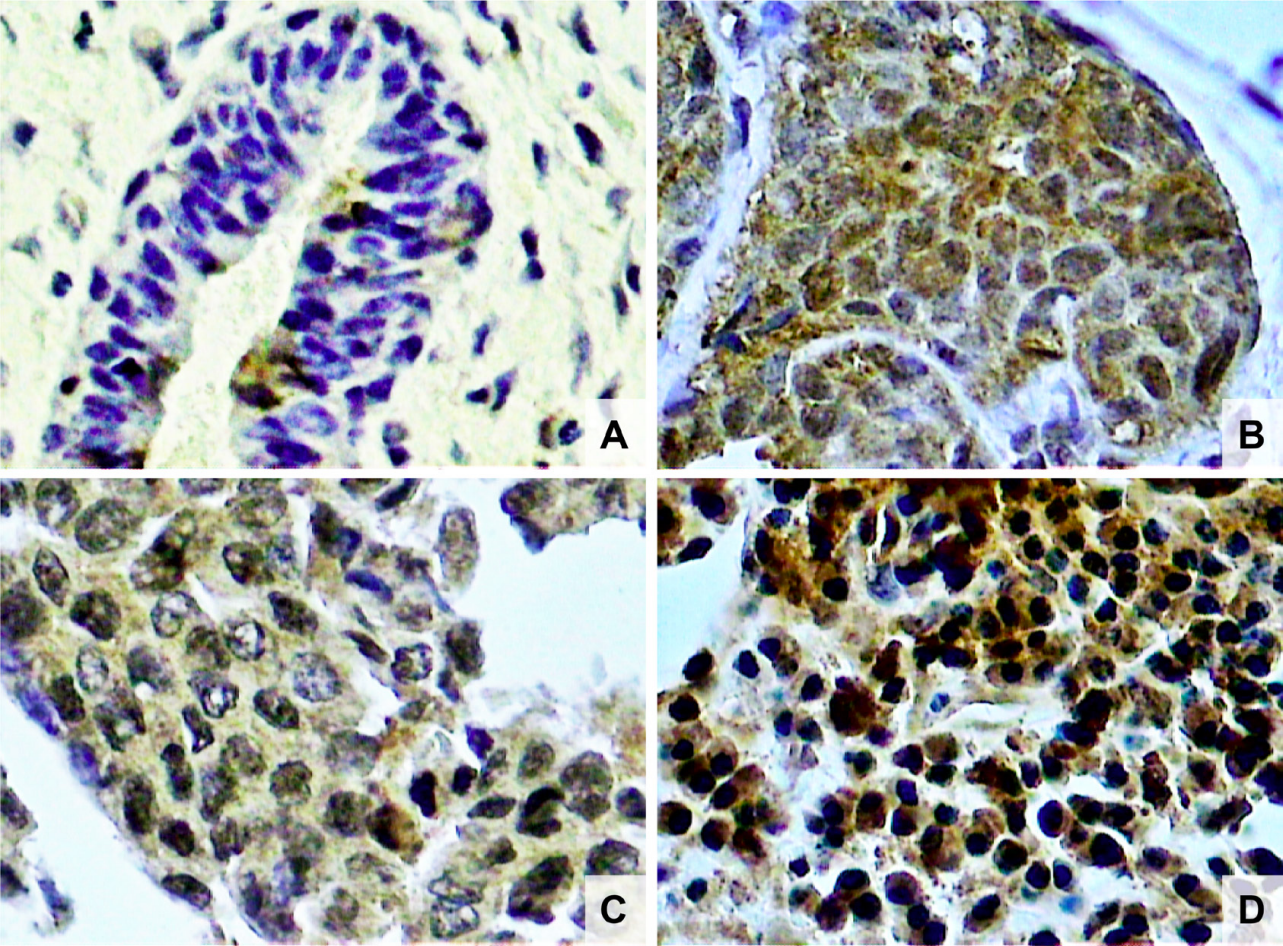
Photomicrographs of histological sections of breast fibroadenoma (**A**), histological grade 1 breast cancer (**B**), grade 2 breast cancer (**C**) and grade 3 breast cancer (**D**). Note the higher concentration of NF-κB stained nuclei in breast cancer cells compared to fibroadenoma cells, and also a higher concentration of NF-κB stained nuclei in histological grade 3 than grade 1 breast cancer (original magnifcation × 400).

**Figure 3:**
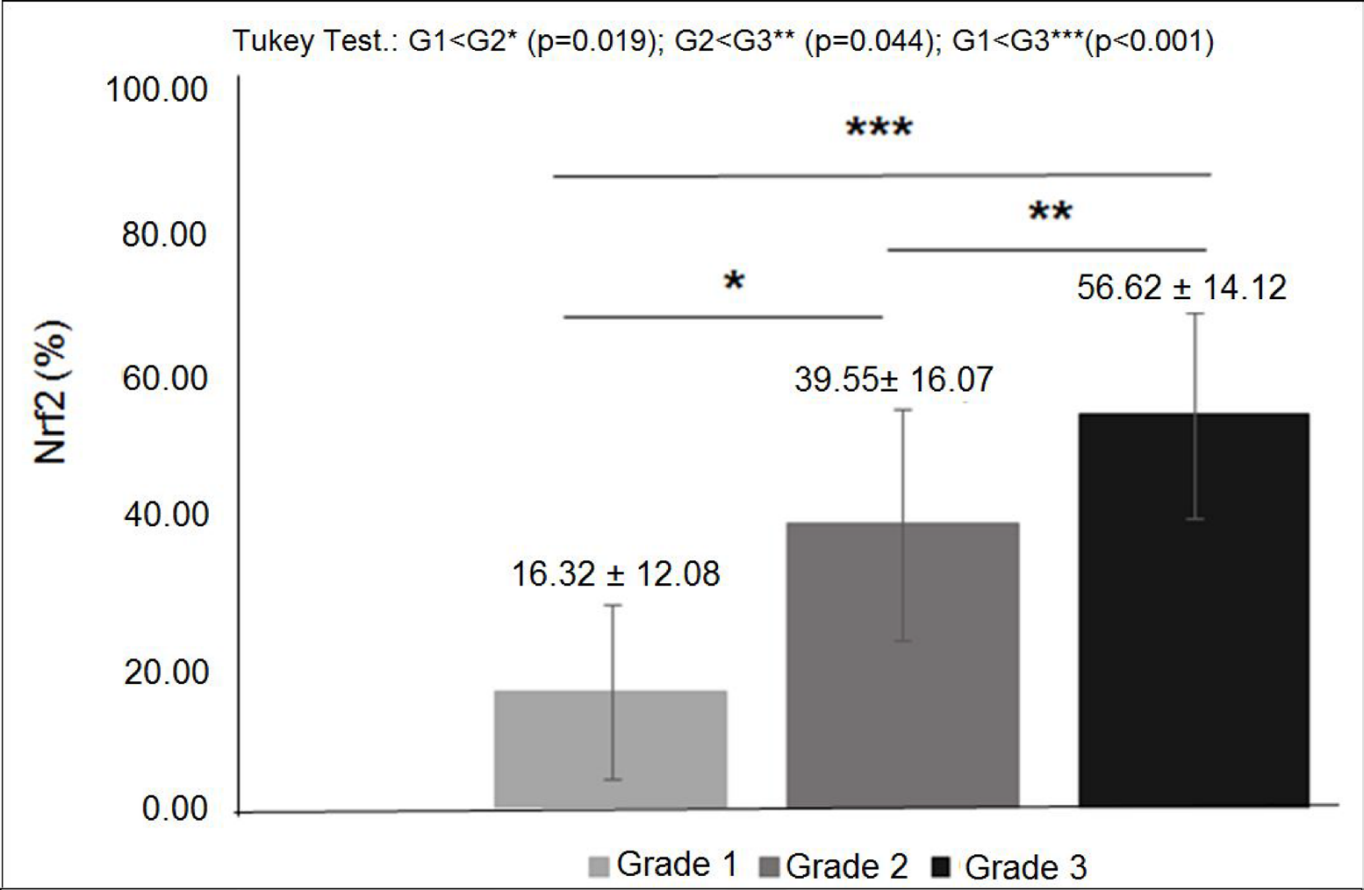
Nrf2 expression in diferent histological grades of breast cancer: mean percentage of Nrf2 expression was significantly higher in histological grade 3 tumors than grade 2 and significantly higher in grade 2 than grade 1 (*p* < 0.05).

**Figure 4:**
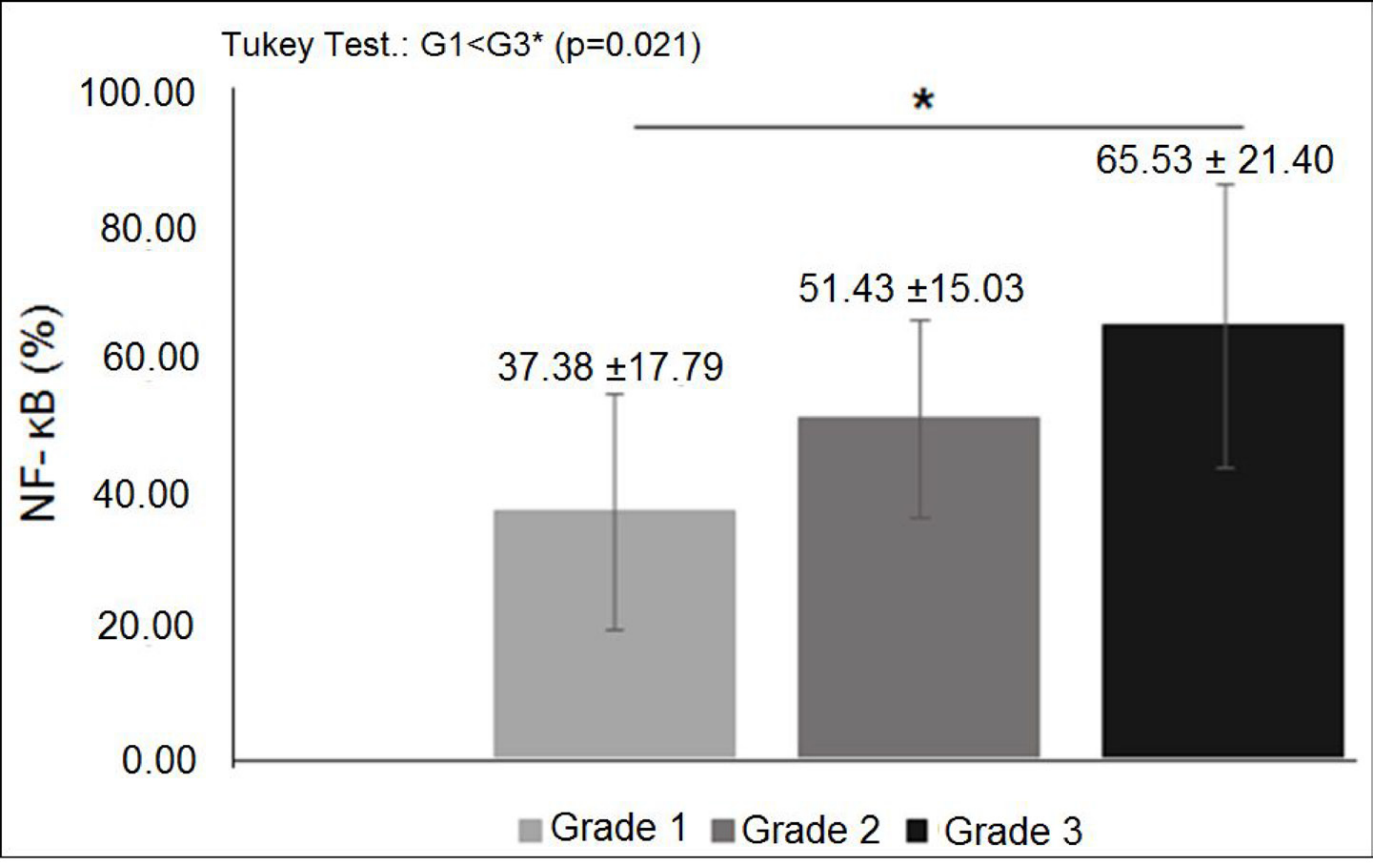
NF-κB expression in diferent histological grades of breast cancer: mean percentage of NF-κB expression was significantly higher in histological grade 3 tumors than in grade 1 (*p* < 0.05).

The characteristics of both groups were homogeneous, except for age and waist circumference (Table 1). Distribution of breast cancer patients according to pathological characteristics showed in relation to the degree of histological differentiation of tumors, a predominance of histological grade 3, as well as the presence of breast tumors larger than 2 cm. Regarding the histological type, invasive ductal carcinoma was present in almost all patients diagnosed with breast cancer. The immunohistochemical profile showed that 50% of women with breast cancer had positive estrogen receptor (ER) and 50% had negative estrogen receptor, and there was a predominance of 56.7% for positive progesterone receptor (PR) and 80% negative for human epidermal growth factor receptor 2 (HER2). In addition, the luminal molecular subtype B was the most frequent, representing 53.3% of cases, followed by the triple negative subtype at 26.7% (Table 2). Light microscopy showed greater concentration of stained nuclei for Nrf2 and NF-κB in the study group compared to the control group (Figures 1 and 2). The mean percentage of stained nuclei for Nrf2 was 7.12 ± 5.20 and 43.21 ± 19.83 in the control and study groups, respectively (*p* < 0.0001). The mean percentage of stained nuclei for NF-κB was 10.75 ± 7.09 and 56.14 ± 21.19 in the control and study groups, respectively (*p* < 0.0001) (Table 3). Furthermore, Nrf2 expression was significantly higher in histological grade 3 tumors than grade 2 and grade 2 significantly higher than grade 1, while NF-κB expression was significantly higher in histological grade 3 tumors than in grade 1 (*p* < 0.05) (Figures 1 and 2). The box plot in Figures 3 and 4 clearly shows an increase in the mean percentage of histological grade 3 tumors than grade 1.

**Table 1:** Patient characteristics

Characteristic	Control group (Fibroadenoma) (*n* = 36) Mean ± DP	Study group (Breast cancer) (*n* = 30) Mean ± DP	*p* value
Age (y)	32.92 ± 9.46	40.37 ± 6.77^*^	0.0011
Menarche age (y)	12.86 ± 1.16	13.67 ± 1.79	0.0820
BMI (kg/m^2^)	24.20 ± 4.61	25.70 ± 3.62	0.1540
WC (cm)	79.40 ± 12.34	83.92 ± 8.92^*^	0.0480

^*^There was a statistically significant differences between control and study groups (*p <* 0.05).

**Table 2:** Distribution of breast cancer patients according to pathological characteristics

Pathological features	Breast cancer	
	*n*	%
Tumour grade		
I	04	13.3
II	12	40.0
III	14	46.7
Tumour size		
< 2 cm	12	40
≥ 2 cm	18	60
Histologycal type		
Ductal *in situ*	01	3.3
Invasive ductal	29	96.7
Hormone receptor status		
ER		
Positive	15	50
Negative	15	50
PR		
Positive	17	56.7
Negative	13	43.3
HER2		
Positive	6	20
Negative	24	80
Molecular subtypes		
Luminal A	03	10.0
Luminal B	16	53.3
HER2+	03	10.0
Triple negative	08	26.7

Abbreviations: ER: estrogen receptor, PR: progesterone receptor, HER2: human epidermal growth factor receptor-type 2.

**Table 3:** Mean percentage of cases with cells expressing Nrf2 and NF- κB in the control group (fibroadenoma) and study group (breast cancer)

	Control group (Fibroadenoma) (*n* = 36) Mean ± DP	Study group (Breast cancer) (*n* = 30) Mean ± DP	*p* value
Nrf2	7.12 ± 5.20	43.21 ± 19.83^*^	0.0001
NF-κB	10.75 ± 7.09	56.13 ± 21.19^*^	0.0001

^*^There was a statistically significant differences between control and study groups (*p <* 0.05).

## DISCUSSION

There is evidence that Nrf2 and NF-κB are good biomarkers due to their high expression in invasive breast carcinoma cells. However, few studies have attempted to elucidate the behavior of these proteins in fibroadenoma, a benign tumor that does not increase the risk of developing breast cancer [15, 16]. This feature makes fibroadenoma an ideal control for determining the expression effect of these proteins on breast cancer and the prognosis in breast cancer.

In the present study, significant overexpression of Nrf2 was observed in breast cancer tissue cells compared to fibroadenoma. To date, only Onodera et al. have investigated Nrf2 expression in human breast fibroadenoma [16]. These authors evaluated 24 women with benign breast lesions, including papilloma, sclerosing adenosis, usual ductal hyperplasia, and fibroadenoma, and found that Nrf2 was positive only in one case of fibroadenoma (4%) while it was positive in 44% of the women with breast carcinoma. Furthermore, the authors showed that the status of Nrf2 immunohistochemistry in women with mammary carcinoma correlated positively with histological grade, Ki-67 labeling index, p62 immunoreactivity, nicotinamide adenine dinucleotide cofactor NAD(P)H immunoreactivity, and quinone oxidoreductase 1 (NQO1). Similarly, in our study, Nrf2 activation was significantly associated with high histological grade. Therefore, Nrf2 overexpression may be an adverse prognostic factor for both relapse and disease-free survival of patients.

NF-κB overexpression was also significantly observed in breast cancer tissue cells compared to fibroadenoma. Based on our literature search, only Sarkar et al. has previously investigated the expression of NF-κB in human breast fibroadenoma [15]. According to these authors, NF-κB was undetectable in control group breast tissue patients, whereas activation of NF-κB was significantly correlated with high grade, large tumor size, high NPI value, ER negativity, PR negativity and HER-2/neu positivity in breast cancer patients.

Overexpression of NF-κB implies aggressive tumor biology in breast cancer and may predict tumors with a likely unfavorable prognosis [15]. In our study, NF-κB activation was significantly associated with high histological grade corroborating the findings of Sarkar et al. and Shapochka, Zaletok and Gnidyuk [15, 17]. Thus, considering that breast cancer in young women commonly has a higher histological grade, as well as an unfavorable hormonal status, and a higher overall mortality rate compared to older women, the application of NF- κB as a biomarker may be a promising target for prognosis and therapy of malignant tumors in women of reproductive age [17, 18].

In our study, it is noteworthy that although the control (fibroadenoma) and study (breast cancer) groups of women were of reproductive age, the average age of women with breast cancer was significantly higher when compared to those with fibroadenoma. Studies show that breast cancer occurs more often in older women, while fibroadenoma is more common in younger women [19, 20]. In addition, the measured waist circumference was higher in women with breast cancer than in those with fibroadenoma, which increases the risk of cancer-related metabolic complications and contributes to a worse prognosis [21].

Based on the results of this study, a new therapeutic approach emerges in breast cancer, since overexpression of Nrf2 and NF-κB is associated with malignant cells with a higher histological grade and, thus, inhibition of expression of these factors may provide a promising strategy in the treatment of these patients. This builds evidence for the need to conduct intervention studies that evaluate the relationship between inhibition of Nrf2 and NF-κB transcriptional pathways and the prognosis of women with breast cancer.

## MATERIALS AND METHODS

### Patients

This study was approved by the Internal Review Board of the Federal University of Piaui (CAAE: 43447015.8.0000.5214). All patients signed a written informed consent form before study initiation. In addition, we confirm that all methods were performed in compliance with current Brazilian laws, in conformity with ethical standards of institutional and national research committees, following the 1964 Helsinki Declaration and its later amendments. A transversal study was carried out, involving 75 premenopausal women with breast tumors. Patients were recruited at the Mastology Clinic of the Getúlio Vargas Hospital, Federal University of Piauí, Brazil, from October 2014 to October 2016. Nine patients were excluded due to technical problems that precluded analysis. Patients were divided into two groups, control group (fibroadenoma, *n* = 36) and study group (breast cancer, *n* = 30). All study participants underwent a specialized surgical procedure for histologic and immunohistochemical confirmation of the tumor. The study included premenopausal patients with levels of follicle-stimulating hormone (FSH) < 30 mUI/ml, fibroadenoma or carcinoma of the breast and no previous oncologic treatment.

### Immunohistochemistry

Breast tissue samples fixed in buffered formalin were cut into 3 μm-thick sections. Sections were deparaffinized in xylol for 5 minutes, dehydrated in absolute ethanol, washed in buffered saline solution at pH 7.4 for 5 minutes and then treated for 5 minutes with 3% hydrogen peroxide (H2O2) in buffered solution to block endogenous peroxidase activity. For antigen retrieval, the slides were placed in racks containing 0.21% citric acid (pH 6.0) and heated in a microwave oven for 15 minutes at maximum power. The slides were cooled, and phosphate-buffered saline was added for a cooling period of 20 minutes. Tissue samples were incubated overnight at 4–8°C with Anti-Nrf2 rabbit polyclonal antibody (1: 100 dilution) and with anti-NF kappa B / p65 rabbit polyclonal antibody (1: 100 dilution), separately. The slides were rinsed with PBS-Tween and incubated with secondary antibody (anti-mouse BA 2000, Vector Laboratories, Burlingame, CA, USA) for 30 minutes at room temperature. After being washed again with PBS-Tween, the slides were incubated with reagents from the ABC Elite detection system (PK 6100, Vector Laboratories) for 45 minutes at room temperature. The samples were rinsed once more with PBS-Tween and incubated with DAB (1.0 ml EnVision FLEX DAB for one drop of chromogen) and for 5 minutes. Finally, the slides were washed with distilled water, counterstained with hematoxylin, stained with ammoniacal solution, dehydrated with absolute ethanol, passed through Coplin jars containing xylol and mounted in Permount resin. Cells expressing Nrf2 and NF-κB proteins were identified by dark brown staining in the nucleus.

### Quantitative method

Two observers, who were blinded with respect to the patients’ identity and had no previous knowledge of any of the cases, performed quantification. It was carried out using a light microscope (Nikon Eclipse E-400, optical microscope, Tokyo, Japan) connected to a color video camera (Samsung digital camera CHC-370 N, Seoul, Korea), which captured the image and transmitted it to a computer equipped with the Imagelab® software program, version 2.3 (Softium Informatica Ltda, Sao Paulo, Brazil) for image analysis.

To determine Nrf2 and NF-κB expressions, we counted nuclei of stained cells under a microscope with a magnification of 400X. At least 600 cells of the breast epithelium were counted on each slide, in random fields, starting in the area of highest Nrf2 and NF-κB concentration in the cell nucleus, using Processing Software and Image Analysis-Image Lab® (SOFTIUM Informatica Ltda, São Paulo, Brazil). The percentage of stained cells for each case was obtained from the ratio between the number of cells with stained nuclei and unstained nuclei multiplied by 100 (labelling index).

### Statistical analysis

Data were analyzed using SPSS statistical program for Windows 18.0. Data were expressed as frequencies, percentages, measures of central tendency and dispersion. The normality of the data was tested with the Kolmogorov-Smirnov test. The Levene test was used to verify data homogeneity. To compare more than two means between normal and homogenous data, we used Student’s t-test and ANOVA. Significant levels were set at *p* values ≤ 0.05.

### Ethics approval

This study was approved by the Internal Review Board of the Federal University of Piaui (CAAE: 43447015.8.0000.5214). All patients signed a written informed consent form before study initiation.
